# Staufen 1 amplifies proapoptotic activation of the unfolded protein response

**DOI:** 10.1038/s41418-020-0553-9

**Published:** 2020-05-15

**Authors:** Mandi Gandelman, Warunee Dansithong, Karla P. Figueroa, Sharan Paul, Daniel R. Scoles, Stefan M. Pulst

**Affiliations:** Department of Neurology, University of Utah, 175 North Medical Drive East, 5th Floor, Salt Lake City, UT 84132 USA

**Keywords:** Cell biology, Neuroscience, Neurological disorders

## Abstract

Staufen-1 (STAU1) is an RNA-binding protein that becomes highly overabundant in numerous neurodegenerative disease models, including those carrying mutations in presenilin1 (PSEN1), microtubule-associated protein tau (*MAPT*), huntingtin (*HTT*), TAR DNA-binding protein-43 gene (*TARDBP*), or C9orf72. We previously reported that elevations in STAU1 determine autophagy defects and its knockdown is protective in models of several neurodegenerative diseases. Additional functional consequences of STAU1 overabundance, however, have not been investigated. We studied the role of STAU1 in the chronic activation of the unfolded protein response (UPR), a common feature among neurodegenerative diseases and often directly associated with neuronal death. Here we report that STAU1 is a novel modulator of the UPR, and is required for apoptosis induced by activation of the PERK–CHOP pathway. STAU1 levels increased in response to multiple endoplasmic reticulum (ER) stressors, and exogenous expression of STAU1 was sufficient to cause apoptosis through the PERK–CHOP pathway of the UPR. Cortical neurons and skin fibroblasts derived from *Stau1*^−/−^ mice showed reduced UPR and apoptosis when challenged with thapsigargin. In fibroblasts from individuals with SCA2 or with ALS-causing TDP-43 and C9ORF72 mutations, we found highly increased STAU1 and CHOP levels in basal conditions, and STAU1 knockdown restored CHOP levels to normal. Taken together, these results show that STAU1 overabundance reduces cellular resistance to ER stress and precipitates apoptosis.

## Introduction

Stress granules (SGs) are cytoplasmic aggregates of ribosome units, mRNA and RNA-binding proteins that assemble as an adaptive response to stress, allowing survival under adverse conditions. Staufen-1 (STAU1) is an RNA-binding protein that localizes to SGs during stress, and can shape a cell’s transcriptome through multiple mechanisms, including regulation of translation efficiency, SG assembly, mRNA transport, and Staufen-mediated mRNA decay [1–11]. We recently identified STAU1 as an interactor of wild-type and mutant ATXN2. Mutant ATXN2 causes the polyglutamine disease spinocerebellar ataxia type 2 (SCA2) [1, 2]. We subsequently discovered substantial increases in STAU1 in multiple cell and animal models of human neurodegenerative diseases, including those carrying mutations in presenilin1, microtubule-associated protein tau, huntingtin, TAR DNA-binding protein-43 gene (*TARDBP*), or C9orf72–SMCR8 complex subunit (*C9orf72*) [1], as well as stroke and myotonic dystrophy [1, 3, 12]. STAU1 overabundance can also be triggered by a variety of acute noxious stimuli such as calcium increase, endoplasmic reticulum (ER) stress, hyperthermia, and oxidative stress [1, 13]. Autophagy defects and IRES-mediated translation are the currently described mechanisms for STAU1 increase, both relevant protein abundance regulatory pathways during neurodegeneration [1, 13].

In neurodegenerative diseases, pathological mutations cause an increased load of misfolded and aggregated proteins and alterations in calcium homeostasis, leading to ER stress and activating the unfolded protein response (UPR) [14, 15]. The UPR is a coordinated cellular response orchestrated by three main signaling pathways downstream of protein kinase RNA-like ER kinase (PERK), inositol-requiring enzyme 1 (IRE1), and activation transcription factor 6 [15]. Activation of the UPR upregulates adaptive mechanisms that promote proper protein folding and regulate calcium balance, including chaperone gene expression, global suppression of protein synthesis, and stimulation of autophagy and the proteasome. Failure to restore ER homeostasis leads to eIF2*α* phosphorylation downstream of PERK and induction of the proapoptotic transcription factor C/EBP homologous protein (CHOP), triggering the intrinsic apoptotic pathway [15–17]. In addition to signaling through the UPR, phosphorylation of eIF2*α* is a critical early step of SG formation, as inhibition of protein synthesis leads to the aggregation of inactive translation complexes into SGs [18, 19].

Here we demonstrate that STAU1 is required for the activation of apoptosis triggered by ER stress. Accordingly, *STAU1* knockout cells were refractory to apoptosis induced by ER stress. STAU1 knockdown was sufficient to prevent the terminal activation of the UPR in cellular and animal models of SCA2 and ALS associated with improvement of motor deficits in vivo [2]. In all, our study describes a novel connection between the RNA-granule protein STAU1 and ER-stress-induced apoptosis that can be targeted in neurological diseases.

## Methods

### Cell lines and cell culture

Cell culture media and reagents were purchased from Thermo Fisher Scientific unless otherwise specified. HEK293 cells and fibroblasts were maintained in DMEM supplemented with 10% fetal bovine serum. Gene editing of endogenous ATXN2 in HEK293 cells to express ATXN2 with 58 CAG repeats was performed with CRISPR/Cas9 according to the published protocols [20], as detailed previously in our work [2]. Cells were periodically screened by PCR to confirm the preservation of ATXN2-Q58. Fibroblasts from patients were obtained from a skin punch biopsy or from Coriell Cell Repositories (Camden, NJ, USA). All subjects biopsied gave written consent and procedures were approved by the Institutional Review Board at the University of Utah (IRB_00035351 and IRB_00040048). Supplementary Table 1 lists all human fibroblasts, their genetic mutation and repository identification number. All mutations were verified by PCR sequencing. Identity authentication of HEK293 cells and human fibroblasts was carried out by short tandem repeat analysis with the GenePrint 24 System (Promega, USA) and mycoplasma testing was carried out regularly.

### Mice

All mice were housed and bred in standard vivarium conditions and experimental procedures were approved by the Institutional Animal Care and Use Committee (IACUC) of the University of Utah. The *Stau1*^*tm1Apa*(−/−)^ (*Stau1*^−/−^) mouse [5] was a generous gift from Prof. Michael A. Kiebler, Ludwig Maximilian University of Munich, Germany. *Stau1*^−/−^ mice were maintained in a C57BL/6BJ background and Pcp2-ATXN2[Q127] (*ATXN2*^*Q*127^) mice [21] were maintained in a B6D2F1/J background. *ATXN2*^*Q127*^ (Pcp2-ATXN2[Q127]) mice [21] were crossed with *Stau1*^*tm1Apa*(−/−)^ (*Stau1*^−/−^) mouse to generate *ATXN2*^*Q127/Tg*^ *Stau1*^*tm1Apa*(+/−)^ and *ATXN2*^*Q127/Wt*^ *Stau1*^*tm1Apa*(+/−)^. These mice were then bred to produce *ATXN2*^*Q127/Tg*^ *Stau1*^*tm1Apa*(−/−)^ and *ATXN2*^*Q127/Wt*^ *Stau1*^*tm1Apa*(−/−)^ in a mixed background of B6D2F1/J and C57BL/6J. Animals were genotyped according to the previously published protocols [5, 22]. To obtain western blot samples, mice were sacrificed at 34 weeks of age.

### Primary cultures of cortical neurons

Cultures of cortical neurons were prepared from WT or *Stau1*^−/−^ neonatal mice euthanized according to the IACUC approved protocols. Cortices from 6 to 7 animals were isolated, cut into 2 mm segments, and incubated with 50 units of papain (Worthington Biochemical, USA) in Earle’s balanced salt solution with 1.0 mM l-cysteine and 0.5 mM EDTA for 15 min at 37 °C. Digested tissue was washed with EBSS and mechanical dissociation was performed with a 1 ml micropipette in the presence of 0.1 mg/ml of DNase1 (Sigma-Aldrich). Cell suspension was filtered through a 40 µm strainer (Corning) to remove any remaining aggregates. Neurons were seeded on poly-l-ornithine (Sigma-Aldrich) and laminin coated plates at a density of 50,000 per cm^2^ in Neurobasal Plus medium containing 2% B27 Plus supplement. To prevent proliferation of glial cells 1 µM cytosine arabinoside (Sigma-Aldrich) was added on day 2 and 90% of the media volume was changed after 24 h. From there on, 60% of culture medium was replenished every 2–3 days. Experiments were conducted on day 9–10 by replacing all culture media with fresh media containing thapsigargin or vehicle (DMSO).

### DNA constructs, siRNA, cell treatments, and transfections

Plasmid construct 3xFlag-tagged STAU1 (3xF-STAU1) was prepared as detailed previously [2]. All constructs were cloned into a pCMV-3xFlag plasmid (Agilent Technologies, USA) and verified by sequencing. The sequences or commercial origin of all siRNAs used in this study are listed in the Supplementary Table 4. For siRNA experiments HEK293 cells or fibroblasts were transfected with lipofectamine 2000 and harvested after 72 h. For overexpression of recombinant proteins in HEK293 cells we utilized lipofectamine 3000 for 4 h and harvested after 72 h. For experiments involving both overexpression of recombinant protein and siRNA, HEK293 cells were transfected as specified for recombinant protein and 24 h later for siRNA. Cells harvested 48 h after the last transfection. Information on the pharmacological agents used to treat cells are listed in Supplementary Table 3.

### Western blotting

Protein homogenates from cultured cells were prepared by scraping cells in phosphate buffered saline and lysing the pellets in Laemmli sample buffer (Bio-Rad), followed by boiling for 5 min. Tissues were manually homogenized with a pestle in extraction buffer (25 mM Tris-HCl pH 7.6, 300 mM NaCl, 0.5% Nonidet P-40, 2 mM EDTA, 2 mM MgCl_2_, 0.5 M urea, and protease inhibitors (Sigma-Aldrich)). After clarification supernatants were mixed with Laemmli buffer and boiled for 5 min. All protein extracts were resolved by SDS-PAGE and transferred to Hybond P membrane (Amersham Bioscience), blocked in Tris-buffered saline 0.1% Tween-20 with 5% skim milk and primary antibody was incubated overnight in this same solution or 5% bovine serum albumin when antibodies were directed against phosphorylated epitopes. Information about all antibodies used in Supplementary Table 2. After incubation with the corresponding secondary antibody signal was detected using Immobilon Western Chemiluminescent HRP Substrate (EMD Millipore) or SuperSignal™ West Pico PLUS Chemiluminescent Substrate (Thermo Fisher Scientific) and photographed with a Bio-Rad ChemiDoc. Analysis and quantification was performed with Image Lab software (Bio-Rad). Relative protein abundance was first normalized against actin band intensity and then expressed as the ratio to the normalized control.

### Immunocytochemistry

Cells were cultured in NUNC Lab-Tek chamber slides, fixed with paraformaldehyde 4% in phosphate buffered saline, and staining was performed according to the previously published protocols [21, 23]. Imaging was performed at the Fluorescence Microscopy Core Facility, a part of the Health Sciences Cores at the University of Utah.

### Quantitative RT-PCR

RNA extraction from cell cultures was performed with the RNeasy mini kit (Qiagen) according to the manufacturer’s instructions and cDNA was prepared from 1 µg of RNA with the ProtoScript cDNA synthesis kit (New England Biolabs). Quantitative RT-PCR was performed at the Genomics Core Facility, a part of the Health Sciences Cores at the University of Utah. PCR reactions were carried out with Sybr Green PCR Master Mix (Thermo Fisher Scientific). Primer sequences are listed in Supplementary Table 4. Gene expression was normalized to GAPDH levels and analyzed with the relative standard curve method.

### Cytotoxicity quantification

Cytotoxicity was quantified with the CytoScan^TM^ LDH Cytotoxicity Assay (G-Biosciences). WT and *Stau1*^−/−^ fibroblasts were plated in 96-well plates and treated with thapsigargin for 24 h. Cell culture supernatant was then collected and LDH was quantified following the manufacturer’s instructions for chemical compound induced cytotoxicity. Results were normalized for each genotype against a maximum LDH release (cells incubated with lysis buffer) and a spontaneous LDH activity (untreated control cells). Cytotoxicity percentage was then calculated as ((compound treated − spontaneous LDH activity)/(maximum LDH release − spontaneous LDH activity)) × 100.

### Statistical analysis

All results are presented as mean ± standard error of the mean (SEM) unless noted otherwise. Comparisons between groups were made using the Student’s *t* test in OriginPro 2017 software. Level of significance was set at *p* ≤ 0.05. Levels of significance are noted as *p ≤ 0.05, **p ≤ 0.01, and ns = *p* > 0.05, unless otherwise specified.

## Results

### ER stress causes increase in *STAU1* mRNA and protein levels

**Fig. 1:**
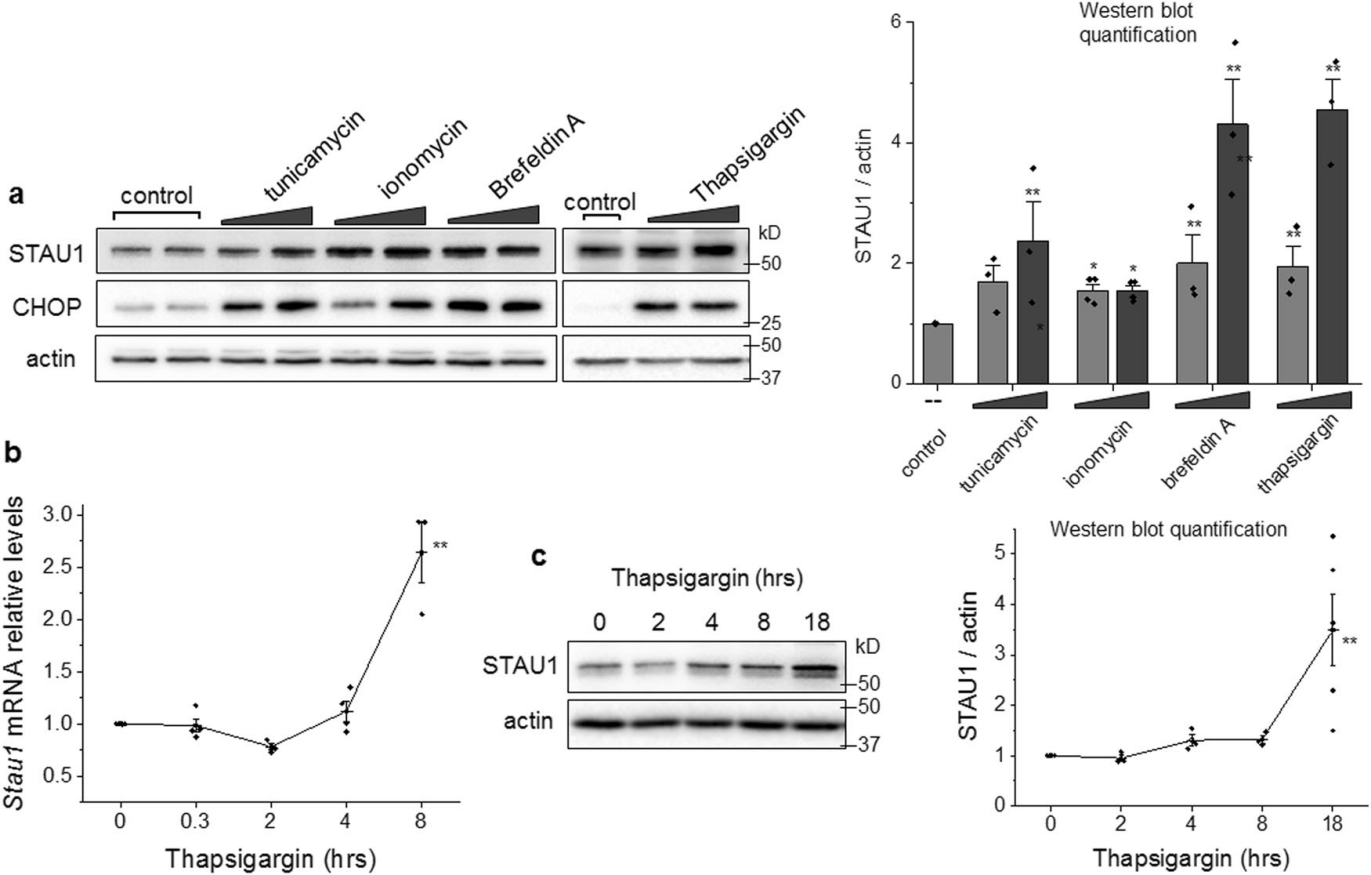
STAU1 overabundance induced by ER stress or calcium dyshomeostasis. **a** HEK293 cells were incubated with tunicamycin (0.1 and 0.5 µM), ionomycin (0.5 and 1 µM), brefeldin A (0.5 and 1 µM), and thapsigargin (0.5 and 1 µM) for 18 h. Levels of STAU1 and CHOP were evaluated by western blot. Graph represents quantification of STAU1/actin from three independent experiments. Single asterisk (*) or double asterisks (**) denote significantly different from untreated control. **b** Relative *STAU1* mRNA levels in HEK293 cells treated with thapsigargin (0.5 µM) for the times indicated. **c** Levels of STAU1 protein in HEK293 cells treated with thapsigargin (0.5 µM) for the times indicated. Graph represents quantification of STAU1/actin for three independent experiments. Data are mean ± SEM. *p < 0.05 and **p < 0.01 according to the paired sample *t* test.

To investigate STAU1 response to ER stress, we treated HEK293 cells with thapsigargin, which depletes ER calcium reserves, tunicamycin to inhibit N-linked glycosylation, ionomycin, a calcium ionophore, or brefeldin A, blocking secretion from the Golgi apparatus [24] and evaluated STAU1 levels and activation of the UPR after 18 h. Increasing doses of thapsigargin, tunicamycin, ionomycin, and brefeldin A resulted in increasing abundances of both STAU1 and CHOP (Fig. 1a). The effects of thapsigargin on *STAU1* mRNA were apparent between 4 and 8 h after treatment and protein levels followed them between 8 and 18 h (Fig. 1b, c).

### Lowering STAU1 protects cells from apoptosis induced by ER stress

**Fig. 2:**
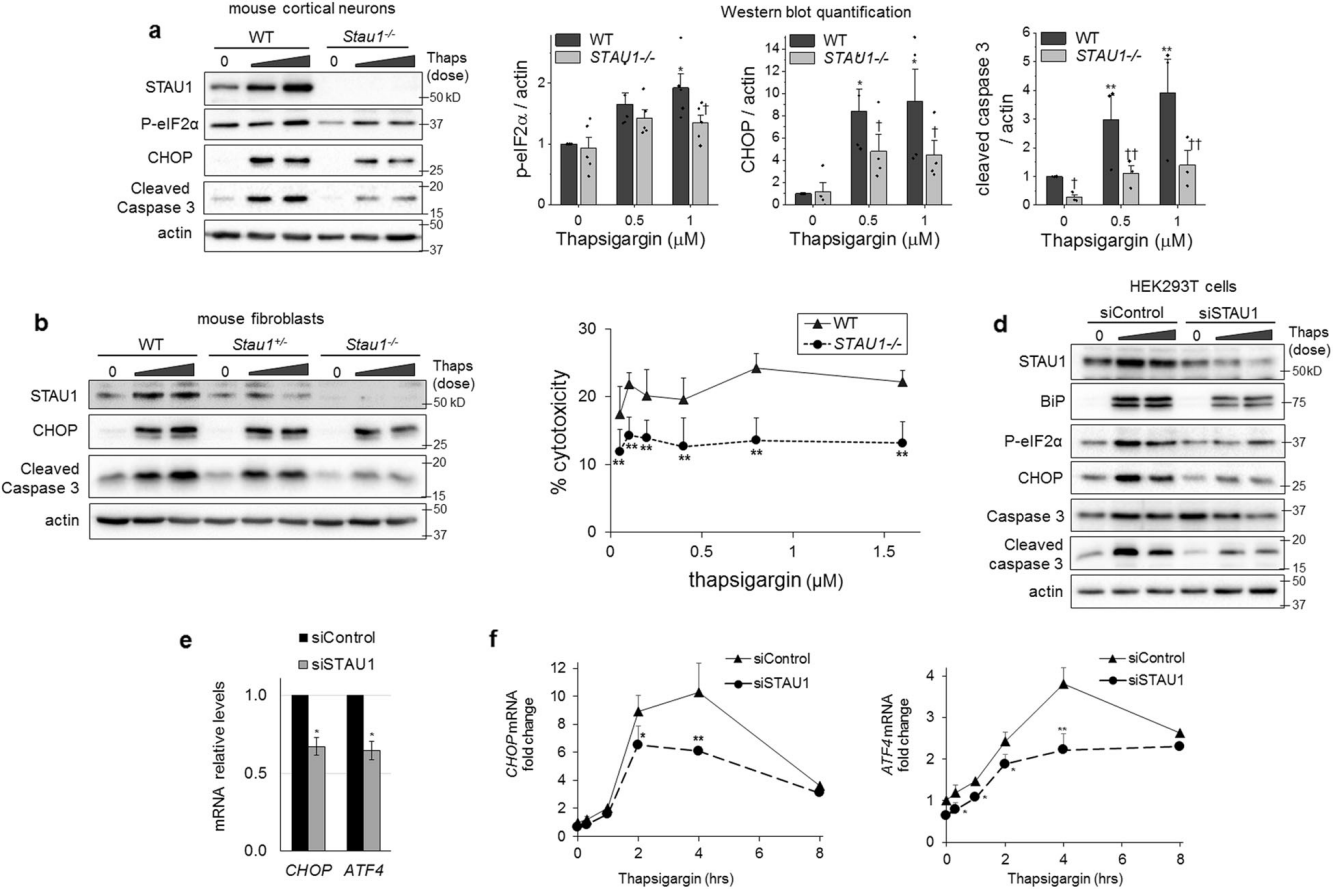
Attenuated UPR and apoptosis in cells deficient in STAU1. Western blots of cultured cortical neurons (**a**) or skin fibroblasts (**b**) from WT, *Stau1*^+/−^, or *Stau1*^−/−^ mice incubated with thapsigargin (0.25 and 0.5 µM, 18 h). Graph represents quantification of target protein/actin from three independent experiments. **c** Cytotoxicity assessment by quantification of LDH release in WT or *Stau1*^−/−^ mouse fibroblasts exposed to indicated doses of thapsigargin for 24 h. **d** Western blots of HEK293 cells transfected with siControl or si*STAU1* for 72 h and incubated with thapsigargin (0.5 and 1 µM) for 18 h. **e** mRNA levels of *CHOP* and *ATF4* in HEK293 72 h post transfection with siControl or si*STAU1* and **f** after treatment with thapsigargin (0.5 µM) for the times indicated. Single asterisk (*), double asterisks (**), single dagger (^†^), or double daggers (^††^) denote significantly different from WT or siControl treated with the corresponding dose of thapsigargin. Data are mean ± SEM. * or ^†^*p* < 0.05, ** or ^††^*p* < 0^.^01 by two-way ANOVA.

In order to study functional consequences of STAU1 overabundance caused by ER stress in neurological disease, we examined the response of mouse primary cortical neurons and skin fibroblasts derived from WT, or *Stau1*^−/−^ mice to thapsigargin. In agreement with the results in Fig. 1, thapsigargin elicited an increase in STAU1 in WT cells, along with large increases in CHOP and cleaved caspase 3. In *Stau1*^−/−^ neurons, CHOP induction was significantly lower (9.3 ± 3 in WT vs 4.6 ± 1.3 in *Stau1*^−/−^ when treated with 1 µM thapsigargin), and cleaved caspase 3 levels were significantly reduced (3.9 ± 1.2 in WT vs 1.4 ± 0.5 in *Stau1*^−/−^) (Fig. 2a). Similar results were seen in fibroblasts, where *Stau*^+/−^ cells evidence a STAU1 dose-dependency of CHOP and cleaved caspase 3 activation (Fig. 2b).

To further demonstrate the role of STAU1 in ER-stress-induced apoptosis, HEK293 cells were transfected with a control siRNA (siControl) or a *STAU1* siRNA (siSTAU1) and challenged with thapsigargin for 18 h. *STAU1* siRNA was effective at preventing induction of STAU1 expression under stress (Supplementary Fig. 2a). Thapsigargin caused a sharp induction of STAU1, UPR, and apoptosis in control cells, whereas the response was greatly attenuated upon silencing of *STAU1*, characterized by lower levels of BiP, p-eIF2*α*, CHOP, and cleaved caspase 3 (Fig. 2d).

To confirm that the role of STAU1 in ER-stress-induced apoptosis was general to ER stress and not specific to thapsigargin, we analyzed cells treated with tunicamycin or brefeldin A, which induce ER stress by vastly different mechanisms. We found that STAU1 knockdown also attenuated the UPR and apoptotosis (Supplementary Fig. 1a, b), indicating STAU1 has a role modulating life and death decisions when cells are faced with ER stress.

Assessment of cell death by quantification of LDH release caused by loss of plasma membrane integrity showed a 63.9% reduction in cytotoxicity in *Stau1*^−/−^ fibroblasts when compared with WT (Fig. 2c) (20.9 ± 2.8 average cytotoxicity in WT vs 13.2 ± 3.2 in *Stau1*^−/−^). These results indicate that the difference in active caspase levels effectively translates to protection against cell death in *Stau1*^−/−^ cells.

*STAU1* silencing significantly reduced baseline levels of *ATF4* and *CHOP* mRNA in HEK293 cells (Fig. 2e), and attenuated their induction by thapsigargin (Fig. 2f). Baseline levels of apoptotic factors were also significantly decreased after *STAU1* silencing (Supplementary Fig. 2b). Their transcriptional induction after thapsigargin did not reach statistical significance (not shown), in agreement with previous reports showing their acute activity is regulated mainly posttranscriptionally.

*ATF4* and *CHOP* transcription increased immediately after addition of thapsigargin and peaked at 4 h (Fig. 2f). In contrast, STAU1 protein and mRNA levels showed a delayed increase, only evident 4–8 h after addition of thapsigargin (Fig. 1b, c). The fact that silencing *STAU1* decreased both basal and induced levels of *ATF4* and *CHOP* mRNAs even before overabundance of STAU1 was evident suggests that baseline levels of STAU1 may play a role in the modulation of *ATF4* and *CHOP* mRNA levels. Therefore, overabundance of STAU1 might not be necessary to mediate its proapoptotic effects.

In all, our data show that STAU1 amplifies the activation of the UPR in a proapoptotic manner and knockdown or knockout of STAU1 is sufficient to prevent apoptosis during ER stress.

### STAU1 causes ER stress and apoptosis through the PERK–CHOP pathway

**Fig. 3:**
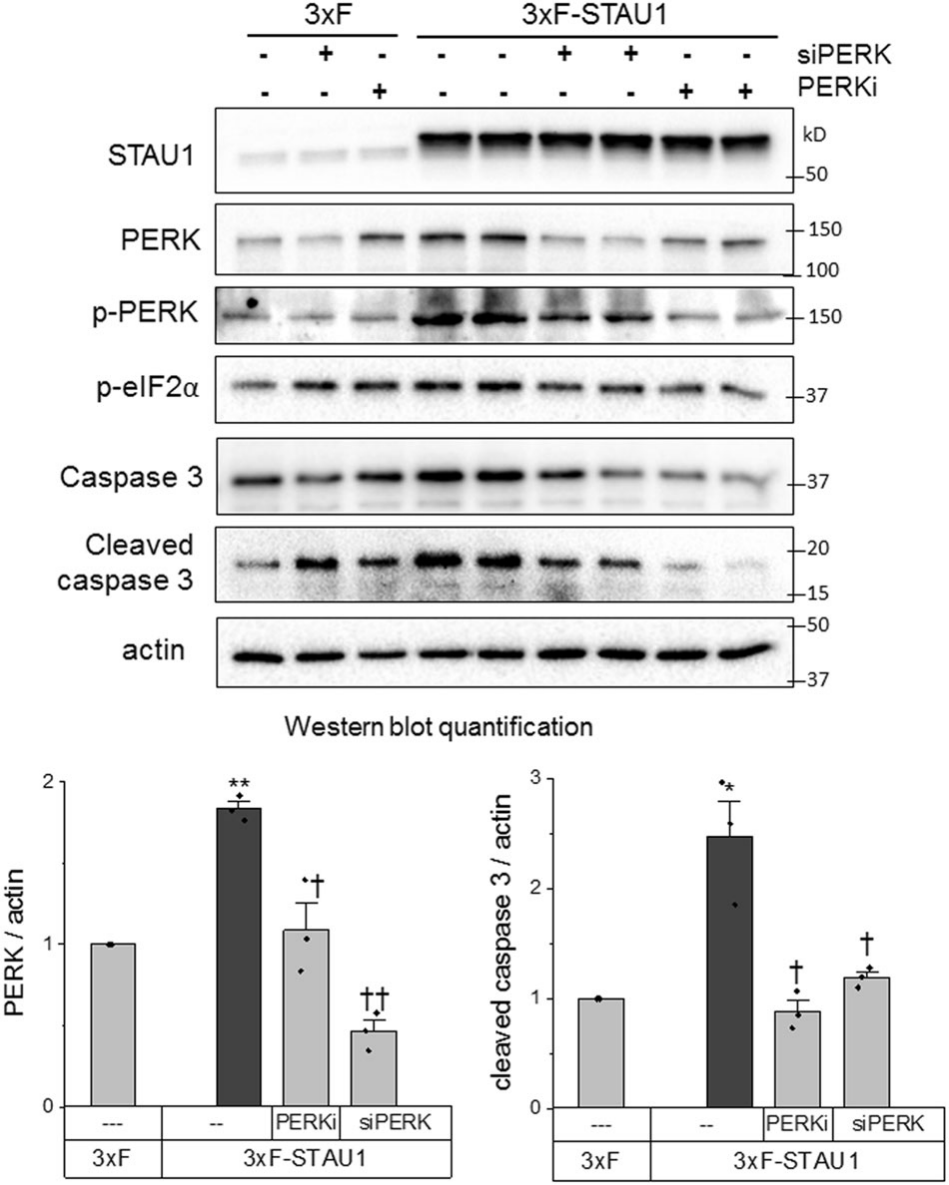
Exogenous STAU1 induces apoptosis through the PERK pathway of the UPR. HEK293 cells were transfected with 3xFlag-STAU1 (3xF-STAU1) or empty vector control (3xF) with addition of siRNA directed at *PERK* (si*PERK*) after 24 h or the PERK inhibitor GSK2606414 (0.5 µM) after 48 h. After a further 18 h, protein levels were analyzed by western blot. Graphs represent the quantification of three independent experiments. Single asterisk (*) or double asterisks (**) denote significantly different from 3xF control. Single dagger (^†^) or double daggers (^††^) denote significantly different from same genotype control. Data are mean ± SEM. ∗ or ^†^*p* < 0.05, ∗∗ or ^††^*p* < 0^.^01 according to the paired sample *t* test.

To understand the pathways by which STAU1 can modulate ER-stress-induced apoptosis, we studied cells expressing exogenous STAU1 in the absence of any other stressors or disease-related mutations. Exogenous STAU1 expression caused a substantial increase in the eIF2*α* kinase PERK and in p-PERK levels, p-eIF2*α* and activation of caspase 3. This was prevented by a PERK inhibitor or siRNA against *PERK* (Fig. 3). These results indicate increased STAU1 signals through the PERK pathway to cause apoptosis, and its inhibition was sufficient to completely prevent the effects of STAU1.

Because STAU1 interacts with the eIF2*α* kinases PKR and GCN2 [25, 26], we studied all four eIF2*α* kinases for involvement in STAU1-mediated apoptosis. We found that each of them contributed to phosphorylation of eIF2*α*, but only PERK and PKR mediated apoptosis (Supplementary Fig. 3). These results indicate that phosphorylation of eIF2*α* is not required for apoptosis triggered by STAU1.

### Decreasing STAU1 prevents ER stress and apoptosis in cellular and mouse models of SCA2

To study if the proapoptotic effects of STAU1 occurred in cells stressed by mutations known to cause neurodegeneration, we studied cells and mice with mutations in ATXN2, the protein mutated in SCA2 [27]. Previously we found calcium dyshomeostasis and increased STAU1 levels in SCA2 cells and mice [2, 28, 29] but we did not study whether this was associated with ER stress. We utilized HEK293 cells edited by CRISPR/Cas9 to introduce an expansion of 58 CAG repeats into one ATXN2 allele (ATXN2-Q58 cells) and the parental HEK293 cell line as control (ATXN2-Q22) [2]. We found that ATXN2-Q58 cells had increased levels of the UPR proteins BiP, IRE1, p-eIF2*α*, spliced *XBP1*, and CHOP. Silencing of ATXN2 was sufficient to restore them to normal levels (Supplementary Fig. 4a, b).

**Fig. 4:**
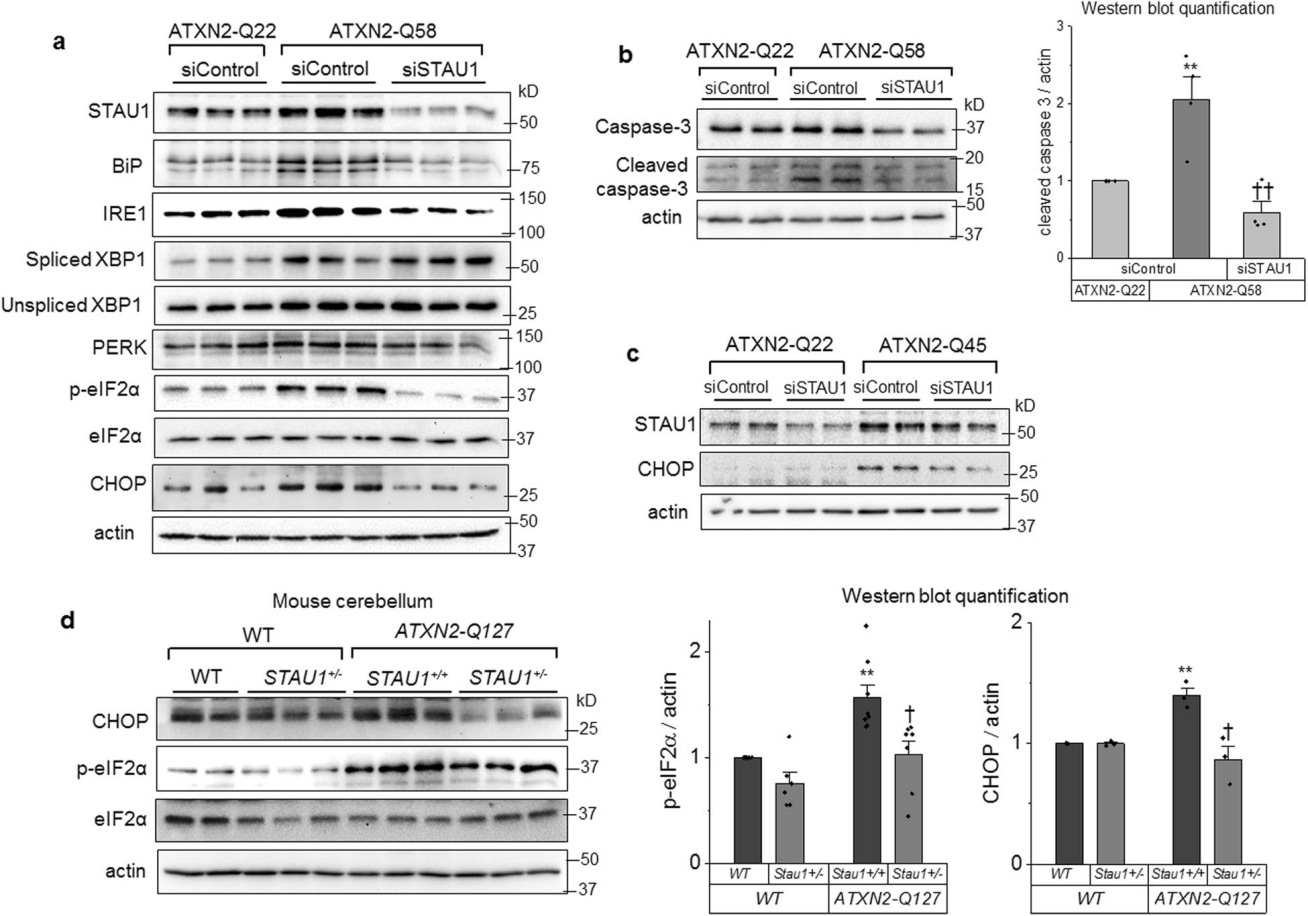
Attenuation of UPR and apoptosis in cellular and animal models of SCA2 by si*STAU1* or genetic interaction. **a** Western blot of proteins involved in the UPR, and **b** caspase 3 and cleaved caspase 3 in ATXN2-Q22 and ATXN2-Q58 cells. Graph represents quantification of three independent experiments. **c** Western blot of fibroblasts derived from an SCA2 patient with ATXN2-Q45 mutation. **d** Western blot of cerebellar tissue from WT, *ATXN2-Q127* mice and *Stau1*^+/−^ haploinsufficient littermates at 34 weeks of age. Graph represents quantification of three animals per genotype. Single asterisk (*) or double asterisks (**) denote significantly different from ATXN2-Q22 or WT control. Single dagger (^†^) or double daggers (^††^) denote significantly different from same genotype control. Data are mean ± SEM. * or ^†^*p* < 0.05, ** or ^††^*p* < 0^.^01 according to the paired sample *t* test.

Mouse models of SCA2 display increased STAU1 levels in the nervous system. Decreasing STAU1 in vivo protects Purkinje neurons and delays the onset of motor symptoms [2]. We studied whether STAU1 linked ATXN2 to apoptosis in models of SCA2. In ATXN2-Q58 cells, *STAU1* silencing was sufficient to decrease UPR activation, evidenced by a significant reduction in the levels of BiP, IRE1, PERK, p-eIF2α, and CHOP (Fig. 4a). In contrast, abundance of unspliced *XBP1* and spliced *XBP1* was increased by *STAU1* silencing. This may represent a restorative mechanism, as XBP1 is essential to prevent cell death caused by ER stress (Fig. 4a). *STAU1* silencing significantly decreased CHOP, total caspase 3 and cleaved caspase 3, indicating that STAU1 was necessary for proapoptotic activation of the UPR in this model (Fig. 4a, b). Fibroblasts derived from an SCA2 patient with a pathological ATXN2 expansion (ATXN2-Q45) recapitulated these findings, as evidenced by a significant decrease in CHOP when *STAU1* was silenced (Fig. 4c). Increased CHOP and p-eIF2α in cerebella of ATXN2-Q127 mice was improved by *STAU1* haploinsufficiency, indicating STAU1 can also modulate ER-stress-induced apoptosis in vivo (Fig. 4d).

Because alterations in calcium homeostasis have been previously described in SCA2 models [28–32], we investigated whether they had a role in induction of apoptosis by STAU1 in ATXN2-Q58 cells. We found that STAU1 levels in ATXN2-Q58 cells were sensitive to changes in cytoplasmic calcium, as they were normalized by the intracellular calcium chelator BAPTA-AM and a CAMKK2 inhibitor (STO-609) (Supplementary Fig. 5a, b). CAMKK2 is Ca^2+^/calmodulin-dependent protein kinase kinase that is activated in response to an increase in the cytosolic-free calcium. In addition, depleting IP3 levels with lithium and valproic acid or blocking calcium efflux from the ER with IP3R or RyR channel blockers (Xestospongin C, 2-ABP, dantrolene, or DHBP) also decreased STAU1, indicating that both types of calcium channels are involved in raising cytoplasmic calcium in ATXN2-Q58 cells (Supplementary Fig. 5a, b). These results provide evidence that a proapoptotic signaling axis involving calcium alterations, STAU1 and ER stress is active in this model of SCA2.

### Decreasing STAU1 prevents ER stress in cellular models of ALS and FTD

**Fig. 5:**
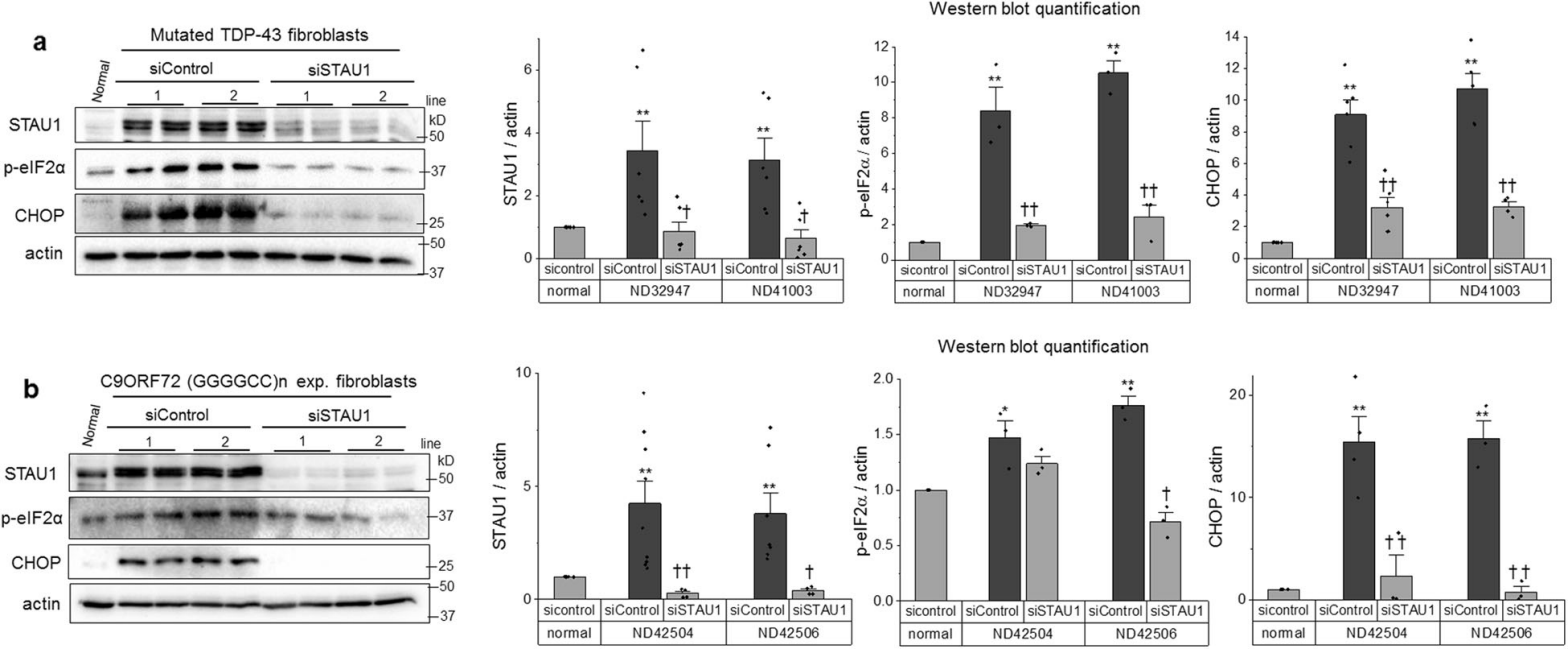
STAU1 knockdown reduces the proapoptotic activation of the UPR in fibroblasts from patients with ALS and FTD-causing mutations. **a** Western blot of STAU1, p-eIF2α, and CHOP in fibroblasts derived from human subjects without disease-related mutation (normal), with *TARDBP* A382T (line 1) and one with *TARDBP* G298S (line 2) (**b**) and two individuals with *C9ORF7*2 GGGGCC repeat expansion (lines 1 and 2). Single asterisk (*) or double asterisks (**) denote significantly different from control patient. Single dagger (^†^) or double daggers (^††^) denote significantly different from same genotype control. Data are mean ± SEM. * or ^†^*p* < 0.05, ** or ^††^*p* < 0^.^01 according to the paired sample *t* test.

We studied fibroblasts derived from two patients with mutations in the TDP-43 gene and two with expansions in C9ORF72, causative of ALS and FTD, respectively. In these cells, STAU1 protein levels were increased between three- and ninefold, along with a markedly activated UPR. Silencing *STAU1* was able to prevent UPR activation, including a strong decrease in CHOP, indicating STAU1 contributed to the pathological phenotype in cells established from ALS and FTD patients (Fig. 5a,b).

## Discussion

STAU1 is an RNA-binding protein with key roles in RNA metabolism and SG formation [2, 11]. Previous reports highlighted a striking overabundance of STAU1 in multiple models of neurological disease [1, 2]. The functional consequences of this observation, however, have not been fully explored. We have identified STAU1 as a modulator of apoptotic signaling during ER stress in multiple models of neurological disease and also in normal cells exposed to pharmacological stressors. This conclusion is substantiated by a number of observations, namely, (a) ectopic expression of exogenous STAU1 caused apoptosis through the PERK–CHOP pathway, (b) STAU1 knockout or knockdown cells showed attenuated UPR and apoptosis in response to ER stressors, and (c) basal levels of UPR activation and apoptosis in cellular and mouse models of SCA2, TDP-43 ALS, and C9ORF72 FTD were markedly decreased by STAU1 knockdown.

Our data suggest that STAU1 lies both upstream and downstream of UPR activation. Exogenous expression of STAU1 was sufficient to induce ER stress, terminally activating the PERK–CHOP pathway (Fig. 3). Analogously, ER stress triggered STAU1 increase, creating a convergent maladaptive feed forward mechanism that amplified STAU1 abundance, ER stress, and apoptosis (Fig. 1).

The PERK–CHOP arm of the UPR is the canonical proapoptotic pathway, orchestrating cell death by inhibiting autophagy, increasing SG formation, altering the redox state of the cell, promoting expression of GADD45 (growth arrest and DNA-damage-inducible protein), and downregulating the antiapoptotic mitochondrial protein BCL-2 [15, 33–36]. These events lead to mitochondrial damage, release of cytochrome c, and activation of caspase 3. In the present study we found that STAU1 could modulate the PERK pathway, upregulating ATF4 and CHOP and therefore precipitating cell death (Figs. 2 and 3).

Absence of caspase 3 cleavage and lowered LDH release were consistent with resistance to ER-stress-induced apoptosis in *STAU1* knockout and knockdown cells (Fig. 2a, b, c). Our results indicate that STAU1 overabundance increases cellular sensitivity to apoptosis, as STAU1 overabundance increased both total caspase 3 and cleaved caspase 3 levels in baseline and stressed conditions, whereas *STAU1* knockout or knockdown decreased baseline transcript levels of *CHOP*, *ATF4*, and *BCL-2* family of apoptosis mediators. As increased caspase 3 levels can decrease the apoptotic threshold when cells are exposed to stress, lowering STAU1 could therefore constitute a strategy to increase resistance to proapoptotic stress by lowering total caspase level and transcripts of *ATF4*,* CHOP*, and *BCL-2* family of apoptosis mediators.

We showed profound changes in eIF2*α* phosphorylation levels in response to STAU1 overexpression (Fig. 3) or *STAU1* silencing (Figs. 4 and 5) and in *STAU1* knockout cells (Fig. 2). A previous report showed that modulating STAU1 abundance did not impact levels of phosphorylated eIF2α under normal or stress conditions, despite being able to alter SG dynamics [6]. A probable reason for this discordance is that in Thomas et al., p-eIF2α was analyzed a maximum of 3 h after the addition of the stress, while we assessed it after 18 h of stimulation or in chronic pathological states generated by disease-causing mutations. In agreement with results in our previous study, knockdown of *Stau1* by only 50% in SCA2 mice significantly reduced the presence of aggregates positive for ATXN2 and STAU1 [2]. Our results suggest that overabundance of STAU1, resulting in p-eIF2α elevations, could contribute to abnormal formation of SG or SG persistence, contributing to aberrant translation, ribostasis, and proteostasis and leading to CHOP-dependent apoptosis [1, 2, 18].

Our study of cells derived from patients with ATXN2, TDP-43, and C9ORF72 mutations, as well as cerebella from SCA2 mice show that the STAU1–CHOP axis described here is basally active in these models of neurodegeneration (Figs. 4 and 5). We previously demonstrated that SCA2 mice benefit from STAU1 knockdown, with improvement of motor and molecular phenotypes and preservation of Purkinje cell firing frequency [2]. Our results suggest that a decrease in ER stress and proapoptotic UPR could be responsible for these phenotypic improvements. In addition, PERK is upregulated in several models of neurodegeneration, including overexpression of TDP-43, prion-related protein and tau, and its inhibition protects against neuronal damage [14, 37]. These data support STAU1 as a preferred therapeutic target for neurological disease compared with PERK, since targeting PERK is limited by its pancreatic toxicity [38–41].

In conclusion, the present work informs on the role of STAU1 in multiple diseases by showing that it is a key modulator of ER-stress-induced apoptosis. STAU1 overabundance caused by ER stress or calcium alterations reduces cellular resistance to ER stress and precipitates apoptosis through the PERK–CHOP pathway. By decreasing ER stress and reducing p-eIF2α, ATF4, CHOP, and caspases, targeting STAU1 could ameliorate proteostasis, ribostasis, and aberrant SG phenotypes in diseases caused by ATXN2, TDP-43, and C9ORF72 mutations as well as other disease gene mutations or sporadic forms of neurodegenerative diseases. Further understanding of the molecular mechanisms linking STAU1 and ER stress will provide insight needed to safely modulate death pathways for therapeutic benefit.

## Supplementary information

supplementary figure legends

supplementary figure 1

supplementary figure 2

supplementary figure 3

supplementary figure 4

supplementary figure 5

supplementary table
