# Supplementary material for: Staufen 1 amplifies proapoptotic activation of the unfolded protein response
Source: Cell Death Differ. 2020 May 15;27(10):2942–51. doi: 10.1038/s41418-020-0553-9 (PMC7492261; doi:10.1038/s41418-020-0553-9)
Supplement: Supplementary file 1 — supplementary figure legends [file 41418_2020_553_MOESM1_ESM.docx]

**Staufen 1 amplifies pro-apoptotic activation of the unfolded protein response**

Mandi Gandelman, Warunee Dansithong, Karla P Figueroa, Sharan Paul, Daniel R Scoles, Stefan M Pulst.

**Supplementary figures and tables**

**
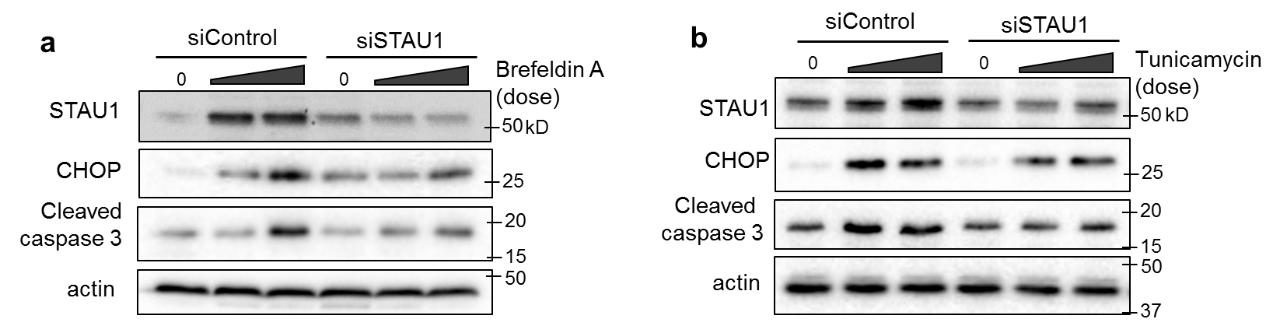
**


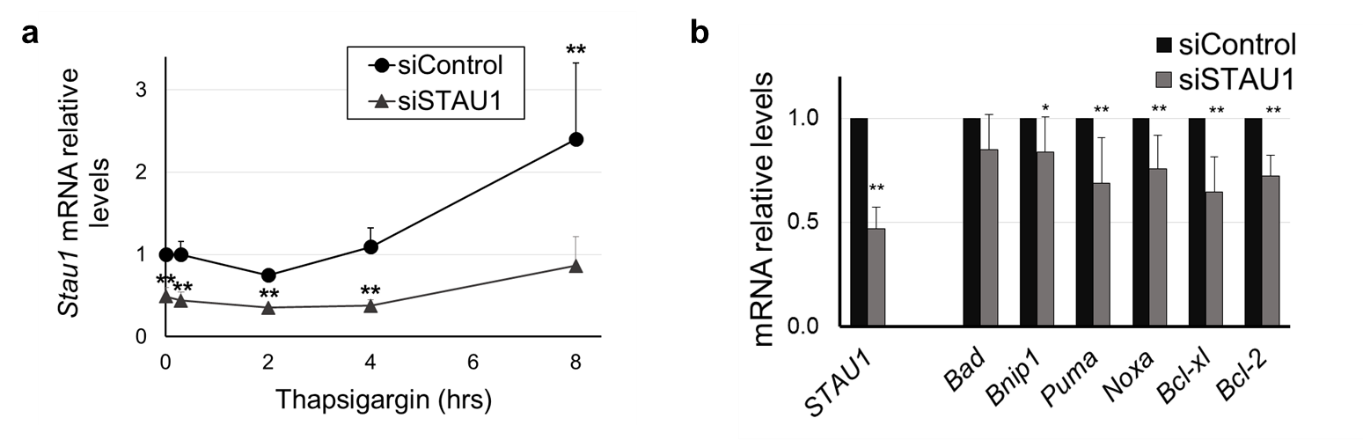


**Supplementary Fig. 2.** **(a)** Relative mRNA levels of *Stau1* in HEK293 cells transfected with siControl or siSTAU1 and treated with thapsigargin after 72hrs. **(b)** Relative mRNA levels of apoptotic factors in HEK293 cells 72 hours after transfection with siControl or siSTAU1. Data are mean ± SEM of at least 3 independent experiments. ^∗^p < 0.05, ^∗∗^p < 0.01 by 2-way ANOVA.

**
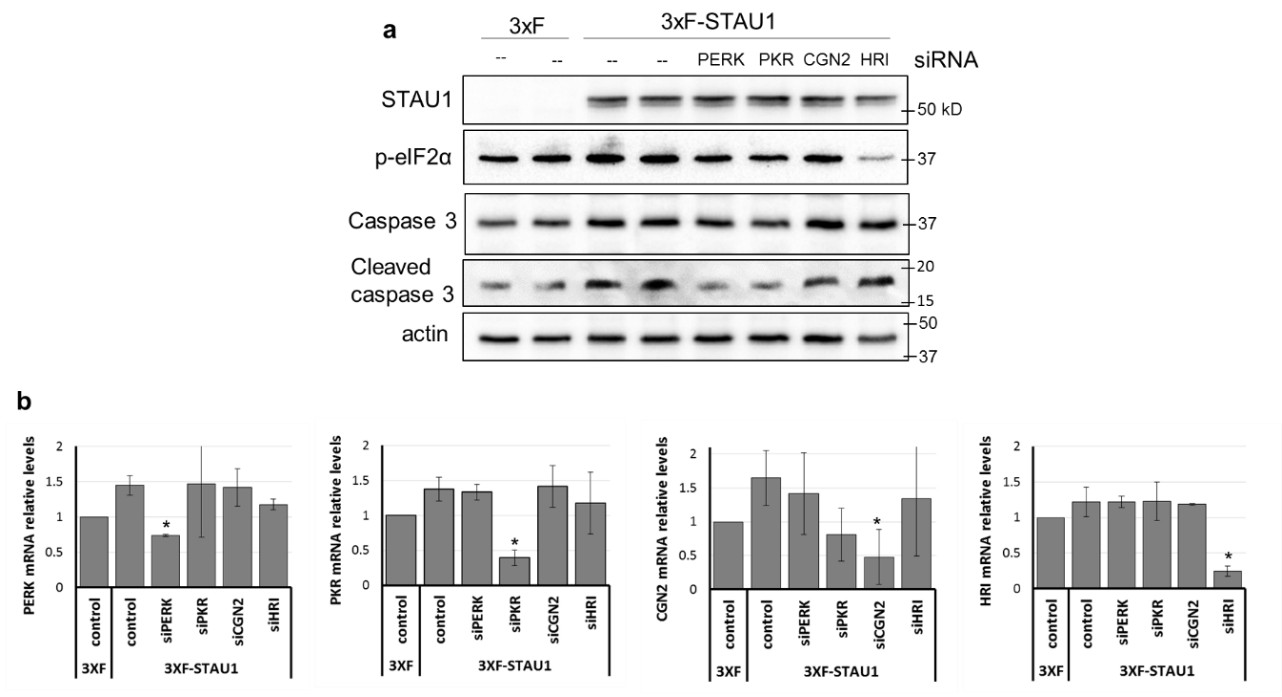

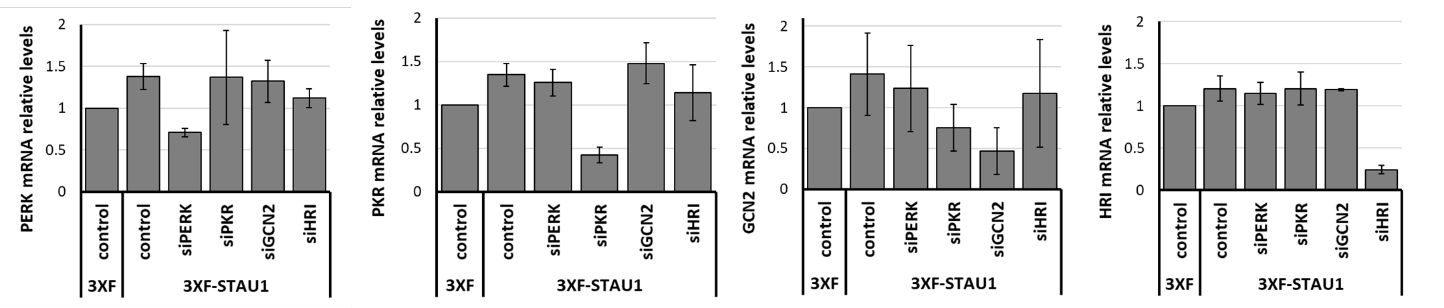
**

**Supplementary Fig. 3. Role of eif2 kinases and p-eIF2α in STAU1-mediated apoptosis**. **(a)** western blots of HEK293 cells transfected with 3xF or 3xF-STAU1, and subsequently with a siControl (--) or the indicated siRNA the day after. **(b)** Relative levels of the indicated genes after transfection as specified in (a).


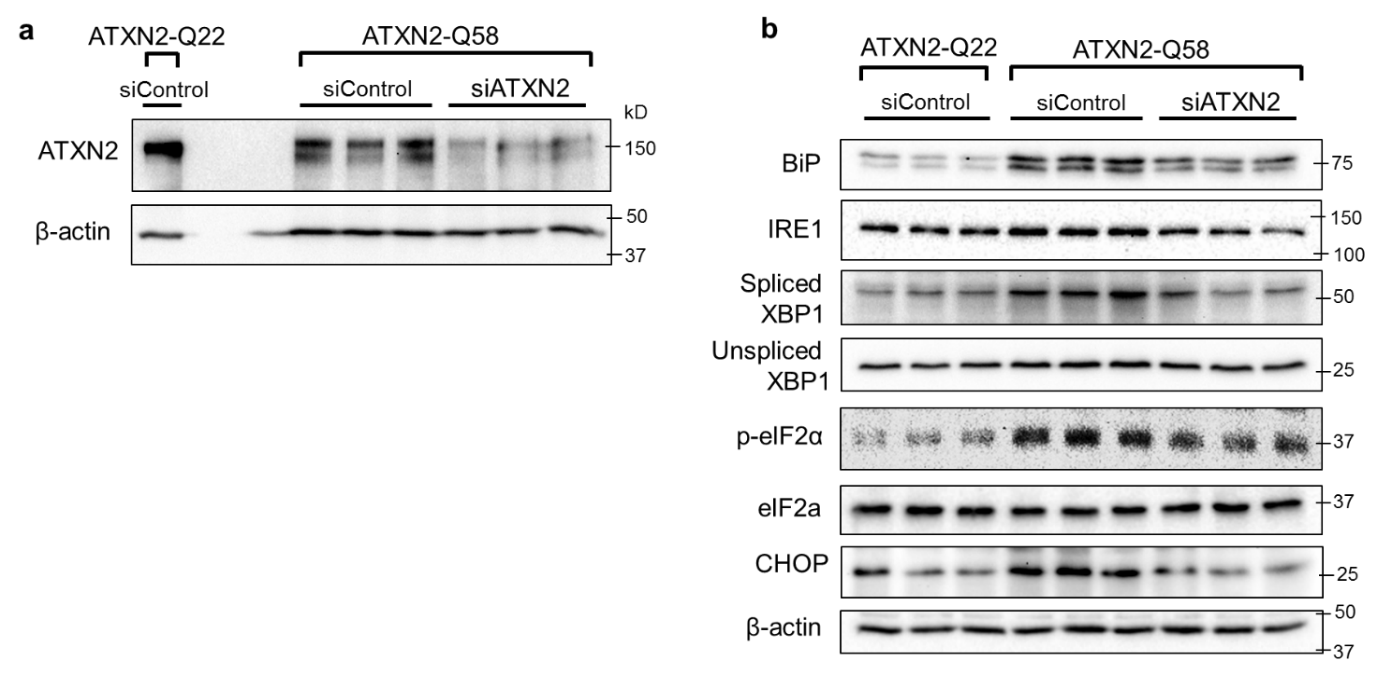


**Supplementary Fig. 4 ATXN2-dependent UPR activation in ATXN2-Q58 cells.** Western blot of **(a)** ATXN2 and **(b)** UPR proteins in HEK293 cells expressing endogenous ATXN2Q22 or ATXN2Q58 cells and ATXN2Q58 cells with siRNA for *ATXN2*.


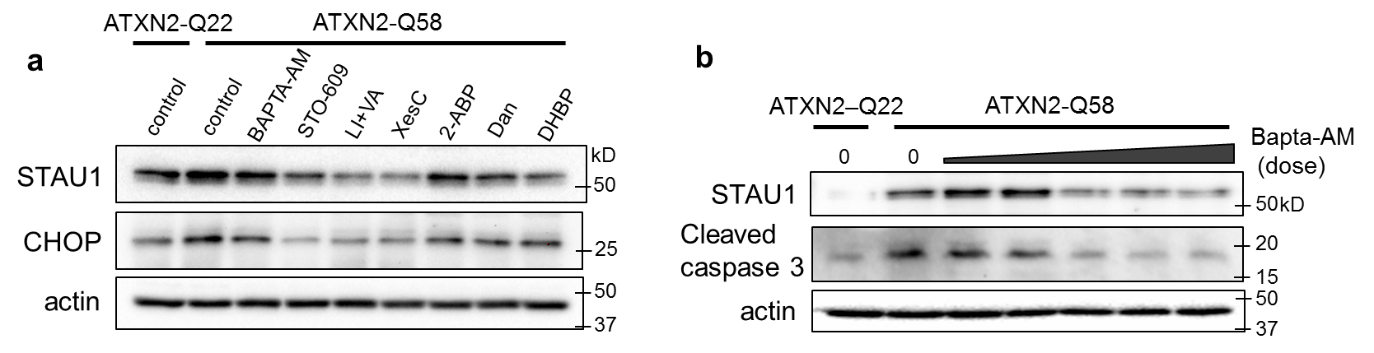


**Supplementary Fig. 5. STAU1 levels are modulated by calcium in ATXN2-Q58 cells (a)** Analysis of STAU1 levels in ATXN2-Q22 or ATXN2-Q58 cells after a 24 hour incubation with the calcium chelator BAPTA-AM (10 µM) CAMKK kinase inhibitor STO-609 (1 µM), IP3 depleting agents lithium + valproic acid (Li+VA, 1mM each), IP3 receptor inhibitors Xestospongin C (Xes C, 0.1 µM) and 2-ABP (1 µM), RyR inhibitors Dantrolene (Dan, 1 µM) and DHBP (1 µM). **(b)** STAU1 levels decrease in response to BAPTA-AM (1, 5, 10, 20 and 30 µM).
