# Supplementary figures and images for: Staufen 1 amplifies proapoptotic activation of the unfolded protein response
Source: Cell Death Differ. 2020 May 15;27(10):2942–51. doi: 10.1038/s41418-020-0553-9 (PMC7492261; doi:10.1038/s41418-020-0553-9)

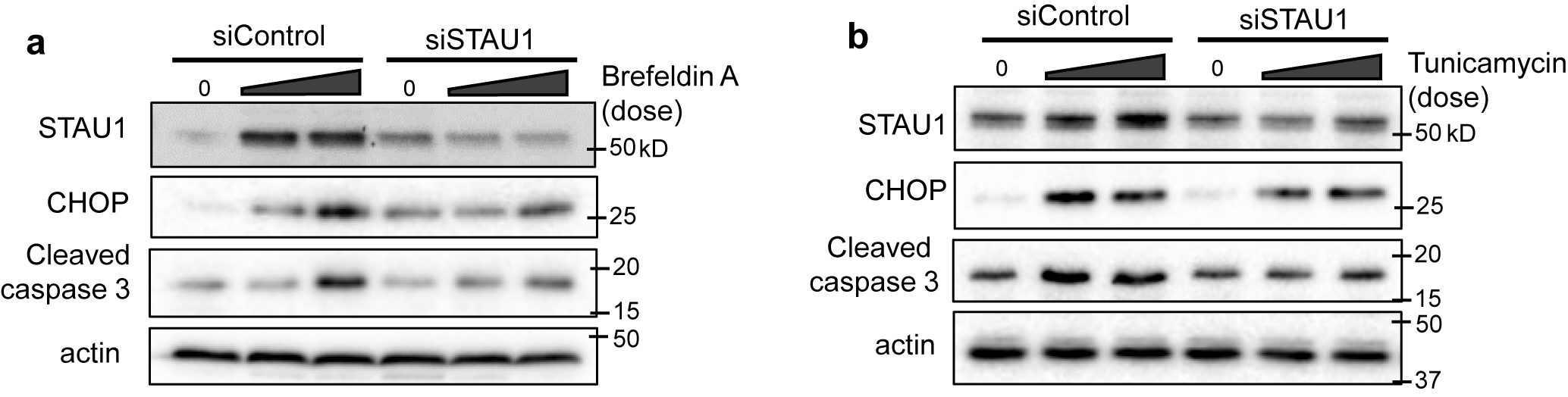

Supplement: Supplementary file 2 — supplementary figure 1 [file 41418_2020_553_MOESM2_ESM.tif]

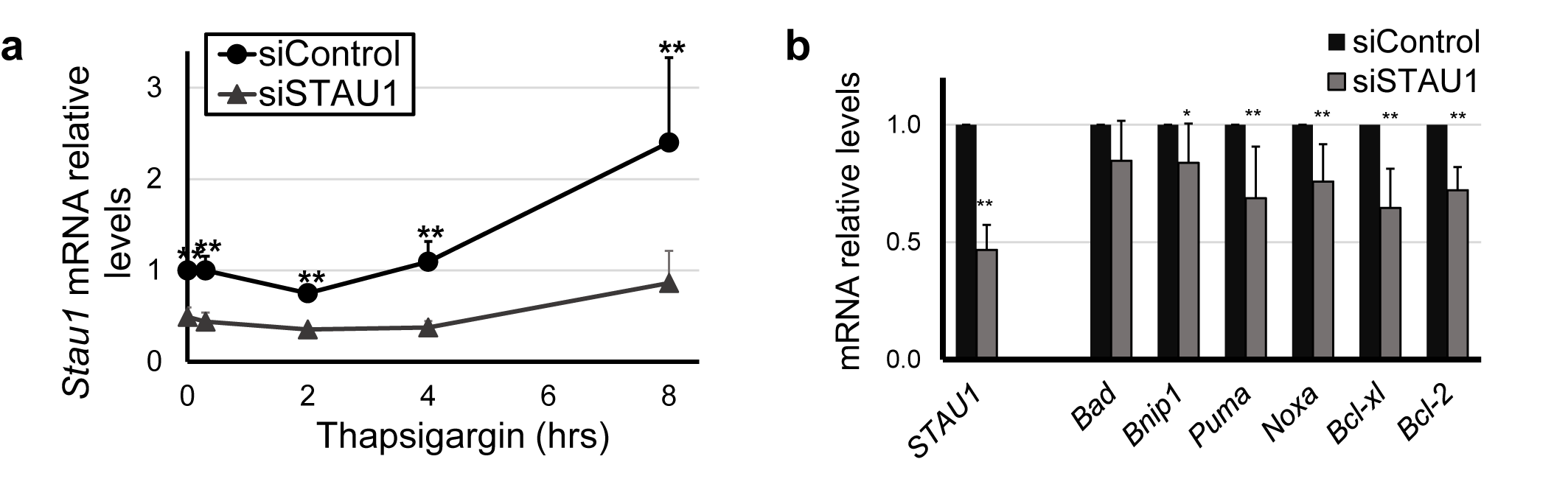

Supplement: Supplementary file 3 — supplementary figure 2 [file 41418_2020_553_MOESM3_ESM.tif]

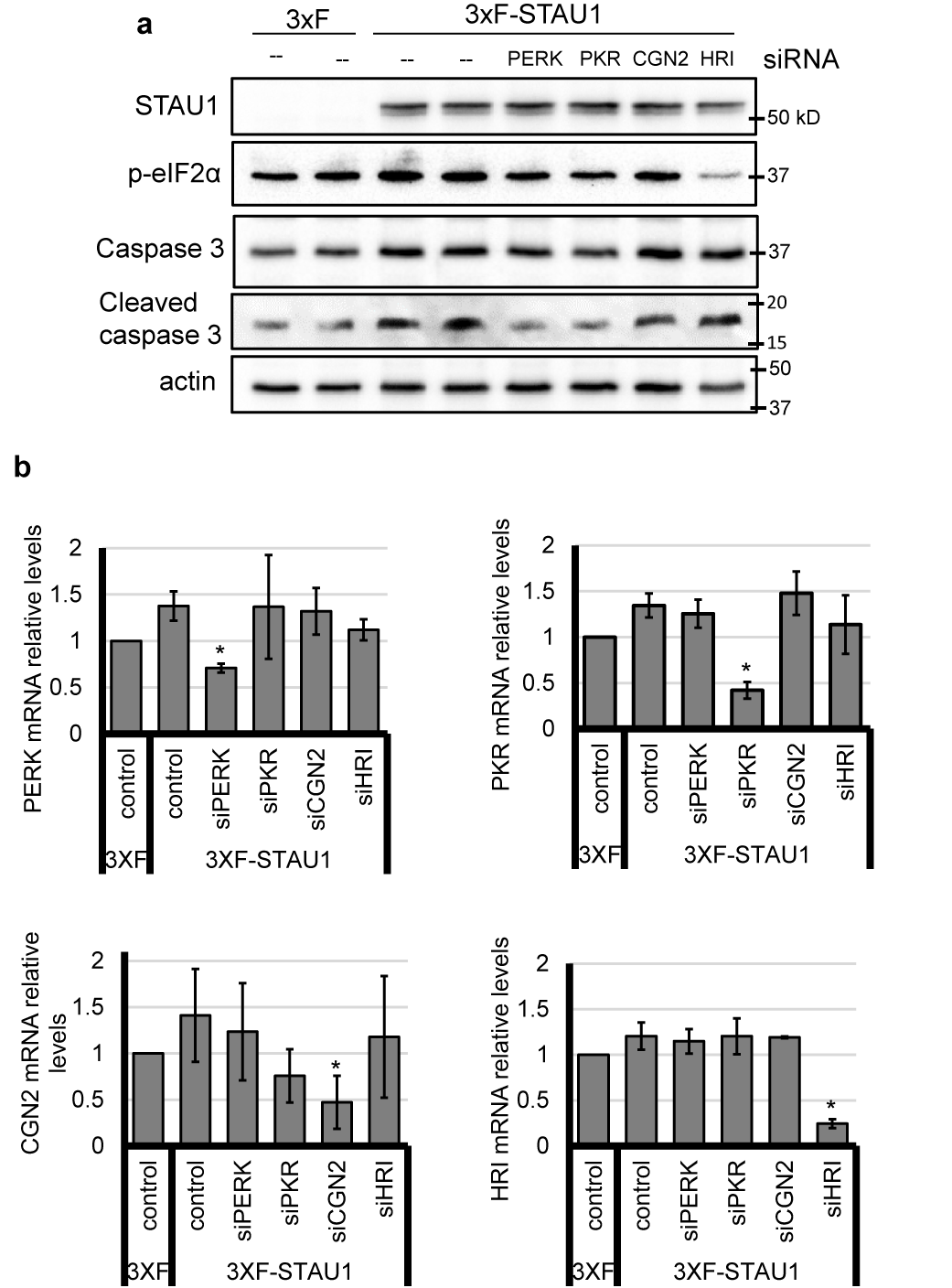

Supplement: Supplementary file 4 — supplementary figure 3 [file 41418_2020_553_MOESM4_ESM.tif]

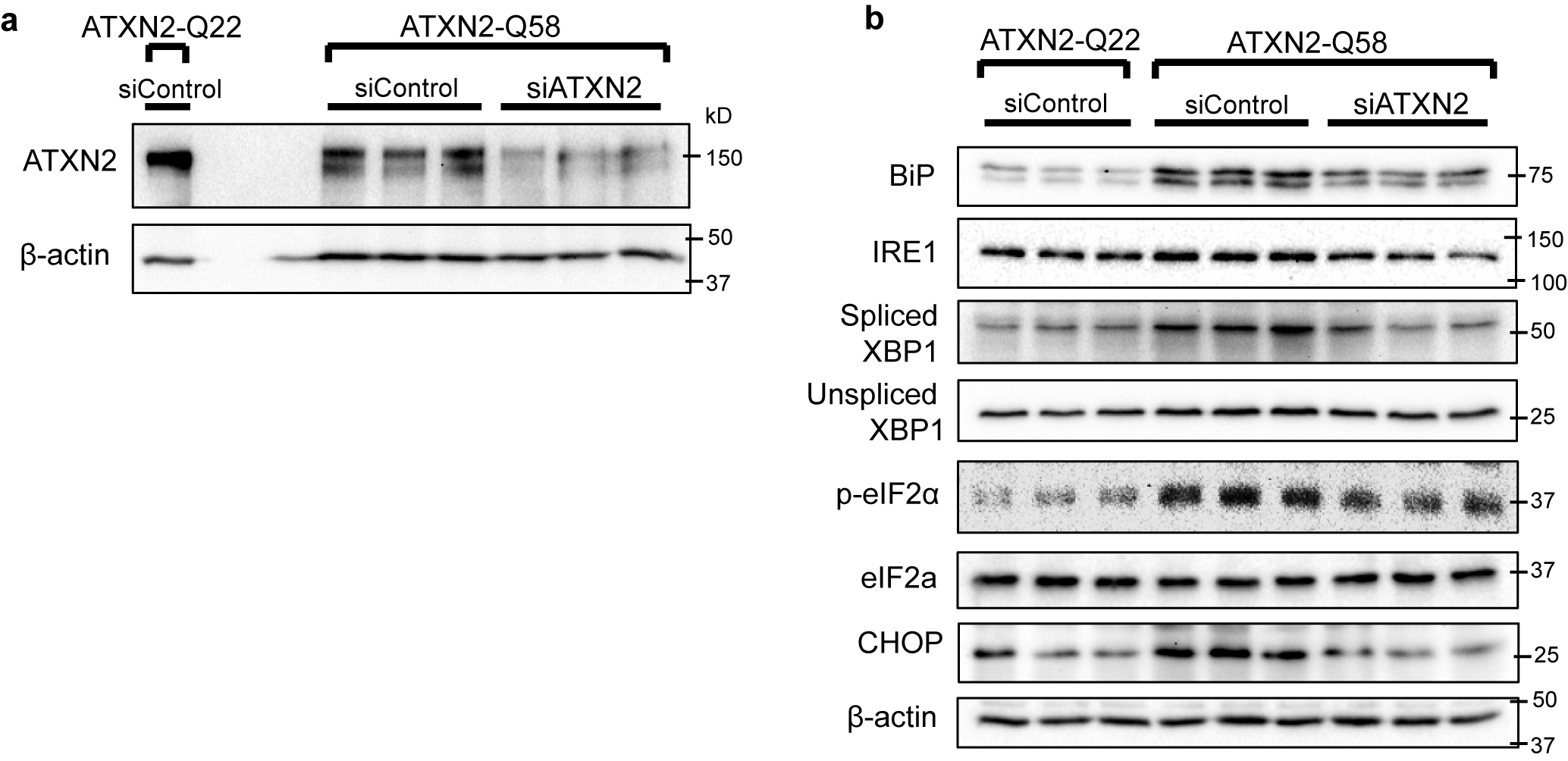

Supplement: Supplementary file 5 — supplementary figure 4 [file 41418_2020_553_MOESM5_ESM.tif]

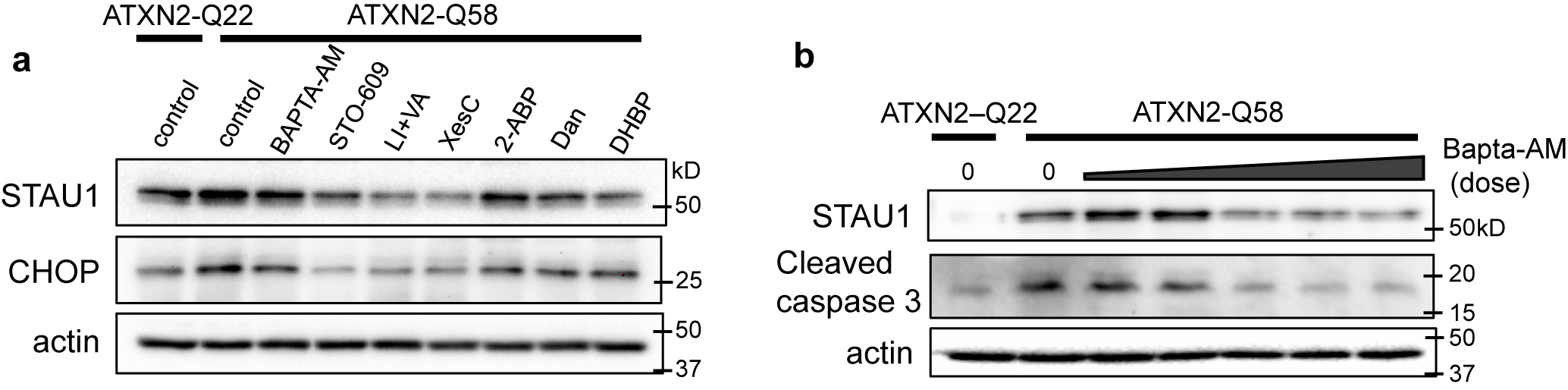

Supplement: Supplementary file 6 — supplementary figure 5 [file 41418_2020_553_MOESM6_ESM.tif]
